# miR-96-5p, miR-134-5p, miR-181b-5p and miR-200b-3p heterogenous expression in sites of prostate cancer versus benign prostate hyperplasia—archival samples study

**DOI:** 10.1007/s00418-020-01941-2

**Published:** 2020-12-17

**Authors:** Kacper Pełka, Klaudia Klicka, Tomasz M. Grzywa, Agata Gondek, Janina M. Marczewska, Filip Garbicz, Kinga Szczepaniak, Wiktor Paskal, Paweł K. Włodarski

**Affiliations:** 1 The Department of Methodology, Center for Preclinical Research, Medical University of Warsaw, 1B Banacha Street, 02-097 Warsaw, Poland; 2 Doctoral School, Medical University of Warsaw, 61 Żwirki i Wigury Street, 02-091 Warsaw, Poland; 3 Department of Immunology, Medical University of Warsaw, 5 Nielubowicza Street, 02-097 Warsaw, Poland; 4 The Department of Pathology, Medical University of Warsaw, 7 Pawińskiego Street, 02-106 Warsaw, Poland; 5 Postgraduate School of Molecular Medicine, 61 Żwirki i Wigury Street, 02-091 Warsaw, Poland; 6 Department of Experimental Hematology, Institute of Hematology and Transfusion Medicine, 14 Indiry Gandhi Street, 02-776 Warsaw, Poland

**Keywords:** Prostate cancer, microRNA, Benign prostate hyperplasia, Laser capture microdissection

## Abstract

MicroRNAs are involved in various pathologies including cancer. The aim of the study was to assess the level of expression of miR-96-5p, -134-5p, -181b-5p, -200b-3p in FFPE samples of prostate cancer, adjacent cancer-free tissue, and benign prostatic hyperplasia. Samples of 23 FFPE prostate cancer and 22 benign prostatic hyperplasias were dissected and HE stained. Compartments of tumor tissue and adjacent healthy glandular tissue were isolated from each sample using Laser Capture Microdissection. Total RNA was isolated from dissected tissues. Expression of miR-96-5p, miR-134-5p, 181b-5p, and miR-200b-3p was determined by real-time RT-qPCR method. The expression of miR-200b-3p was significantly higher in cancerous prostate: both in adenocarcinomatous glands and in the adjacent, apparently unaffected glands compared to BPH samples. The expression of miR-181b-5p was lower in in both prostate cancer tissues and adjacent tissue compared to BPH samples. Expression of miR-96-5p and miR-134-5p was lower in prostate cancer tissues compared to BPH. Levels of miR-96-5p, miR-134-5p, and 181b-5p negatively correlated with the Gleason score. Given further studies, miR-96-5p, miR-134-5p and especially miR-200b-3p and miR-181b-5p may differentiate BPH and PC.

**Electronic supplementary material:**

The online version of this article (10.1007/s00418-020-01941-2) contains supplementary material, which is available to authorized users.

## Introduction

Prostate cancer (PC) is classified as an adenocarcinoma in over 95% cases and preferably locates in the peripheral region of the prostate gland (Oh 2003). PC is the most common cancer in males and accounts for 20% of new cancer diagnoses. Next to lung cancer PC is responsible for the largest number of deaths. It is characterized by a relative high 5-year survival (98%), mainly due to frequent over diagnosis (Siegel et al. 2019). PC over diagnosis is one of the major problems of clinical medicine, that leads to the unnecessary therapy of indolent cancers (Lomas and Ahmed 2020; Costello 2020).

In 1966, Donald Gleason proposed histopathological grading scale for prostatic adenocarcinoma (Gleason 1966). It assesses dominant morphology and the second most common pattern (Oh 2003). It is broadly used as it correlates with prognosis and staging and as well as guides further therapy.

PC usually exhibits indolent growth, however, high-risk or metastatic PC is characterised by 50% recurrence rate. This group of patients requires intensive PSA (prostate-specific antigen) monitoring and/or adjuvant treatment with androgen deprivation therapy (ADT) (Sequeiros et al. 2013). PSA is constitutively produced by prostate gland cells and is used in PC screening tests and as a PC recurrence monitoring marker (Kanwal et al. 2017). Although PSA screening increased detection of PC, results may be biased by non-malignant pathologies, including prostatitis, benign prostatic hyperplasia (BPH) or preanalytical errors, all of which lead to false-positive results. Due to the limited specificity of PSA, novel biomarkers are in demand (Nogueira et al. 2009).

miRNAs are small non-coding molecules which consist of about 18–22 nucleotides. They regulate gene expression by suppressing mRNA translation or affecting mRNA stability in a sequence-specific manner (Aghdam et al. 2018; Kaminska et al. 2018). Therefore, miRNA regulate many aspects of cell biology. In cancer, miRNAs may either suppress tumor growth (tumor suppressor miRs) or promote oncogenesis and tumor-progression (oncomiRs) (Grzywa et al. 2019).

Many authors show an important impact of miRNAs on the pathogenesis of prostate cancer, as well as their role as a diagnostic marker (Kanwal et al. 2017; Sequeiros et al. 2013; Walter et al. 2013b). Recent studies showed miRNAs may serve as diagnostic and prognostic biomarkers in different cancers (Rapado-Gonzalez et al. 2019; Delangle et al. 2019; Butz and Patocs 2019; Bhat et al. 2019). Importantly, miRNAs can be detected in formalin-fixed tissues (FFPE), therefore, they may be potentially an extension of conventional histopathological diagnosis (Klopfleisch et al. 2011; Grzywa et al. 2020). Numerous studies evaluating miRNA expression in PC led to inconclusive results possibly due to the highly heterogeneous structure of the tumor (Yadav et al. 2018; Grzywa et al. 2017). Laser capture microdissection (LCM) overcomes this limitation since it enables to evaluate miRNA expression only within precisely dissected fragments of a sample. We chose four miRNAs that exhibited explicit down- or upregulation in PC in other studies, hsa-miR-96-5p, hsa-miR-134-5p, hsa-miR-181b-5p, hsa-miR -200b-3p (Sequeiros et al. 2013; Janiak et al. 2017; Walter et al. 2013b).

The study aimed to determine miRNA expression in prostates in which cancer has been diagnosed: both in cancerous and morphologically normal, adjacent tissue, as well as in benign prostatic hyperplasia cases from the archival formalin-fixed paraffin-embedded (FFPE) samples.

## Materials and methods

### Archival samples and preparation for LCM

Samples of 23 PC and 22 BPH have been obtained from the Department of Pathology, Medical University of Warsaw. PC patients included previously untreated primary prostate cancer. Each patient with PC underwent a radical prostatectomy in 2014–2020 in the Department of Urology, Medical University of Warsaw. Clinical patients’ data are presented in Table 1. Resected tumors were formalin-fixed and paraffin-embedded according to the standard protocol in the tissue processor. Thereafter the samples were cut on microtome and HE-stained for the pathologist examination (Gleason score assessment). Only fragments with confirmed presence of both neoplastic primary prostate cancer and unaffected prostate gland architecture were included in the study.

**Table 1:** Clinical data of prostate cancer-bearing patients included in study

Case number	Gleason score	Dominant tissue architecture in dissected neoplastic area	Age (years)	Lymphadenectomy	TNM
101	4 + 3	4	60	0	T2b
102	4 + 3	4	65	0	T1c
103	4 + 3	4	60	0	T2b
104	4 + 3	4	65	0	T2c
105	4 + 3	3	63	0	T2c
106	4 + 3	3	76	1	T3a
107	4 + 3	3	73	0	T3b
108	4 + 3	4	73	1	T3a
110	3 + 4	3	61	0	T1c
112	3 + 4	3	59	0	T2c
113	5 + 5	5	69	1	T3b
114	3 + 4	3	66	1	T2c
115	3 + 4	3	63	1	T1c
116	4 + 3	3	68	0	T1c
117	3 + 4	4	50	0	T1c
118	3 + 3	3	68	0	T2c
119	3 + 3	3	57	0	T1c
120	4 + 3	4	67	0	T2c
121	3 + 4	3	71	1	T3a
122	3 + 2	2	74	1	T2a
123	4 + 4	4	61	0	T2a
124	4 + 5	4	70	0	T2c
126	4 + 3	4	59	0	T2c
*N*	23		23	7	23
Mean (± SD)	–		65.31 ± 6.4	–	–
Median	–		65.5	–	T2c

All samples were cut with a microtome to 10 µm slices (Leica, RM2055 model) and were mounted on glass slides (SuperFrost Ultra Plus, Menzel Gläser) with a drop of DNAse/RNAse-free water. Preceding optimization experiments indicated more efficient dissection on SuperFrost® glass slides comparing to dedicated membrane glass slides. Non-membrane slides provided better slices’ adherence and a possibility to dissect sufficient tissue area from surrounding compartments. Then, samples were incubated in a fume hood at 56 °C overnight to increase slices’ adherence. Mounted slices were HE stained according to the standard protocol in a set of stains, alcohol solutions, and xylene. Slides were immediately subjected to LCM.

### Laser capture microdissection

**Fig. 1:**
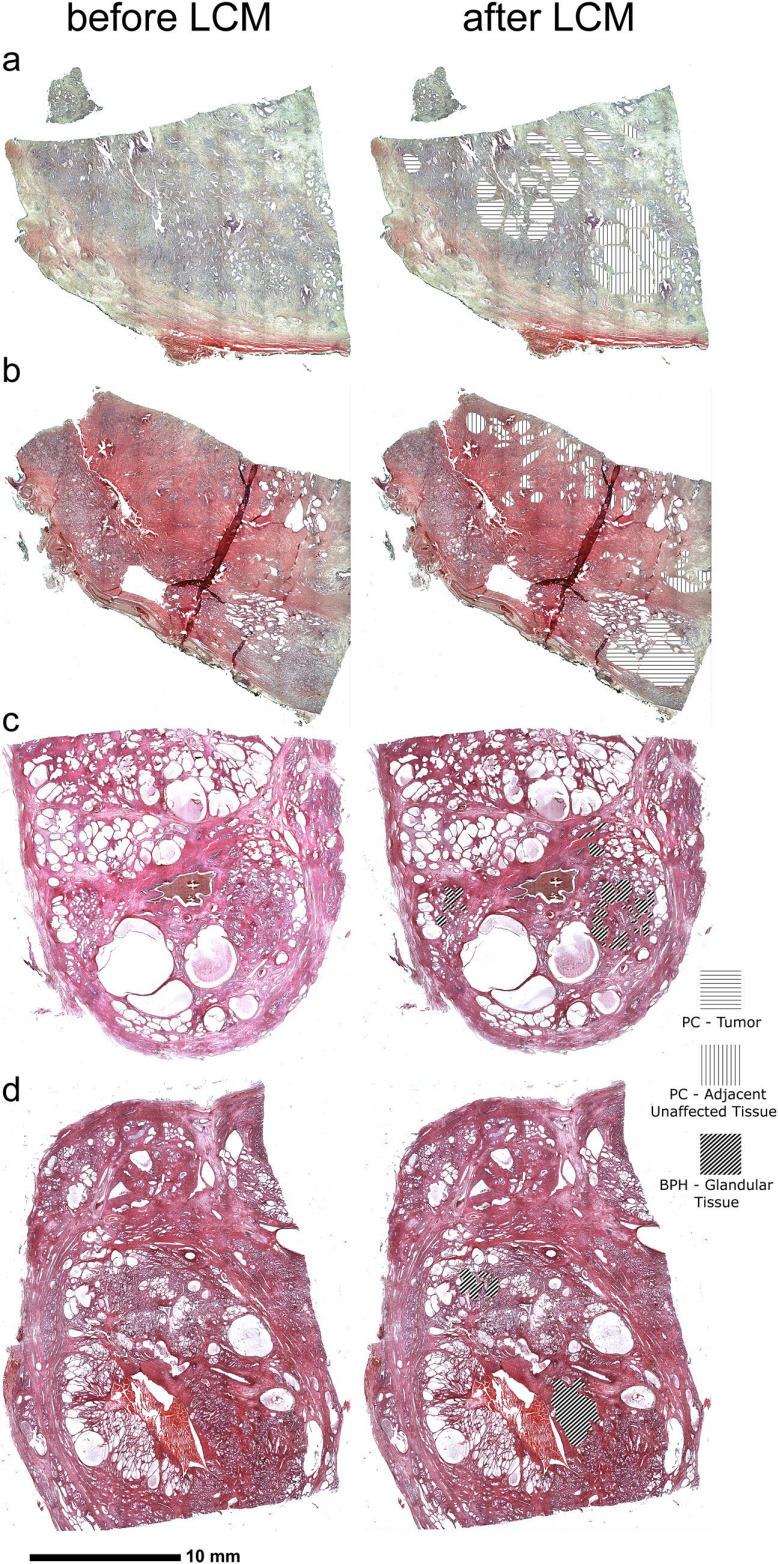
Whole slide images of areas that underwent laser capture microdissection (LCM). Left side of the image represents sections before LCM, right side after LCM. **a**, **b** Samples of prostate cancer (PC). **c**, **d** Samples of benign prostate hyperplasia (BPH). Horizontal lines—tumor area of a PC sample; vertical lines—area of adjacent tissue of PC; diagonal lines—glandular tissue of a BPH sample

Stained and dehydrated sections of PC were subjected to LCM-aided dissection of two regions—engaged by neoplastic process and adjacent tissue that contained only glands of normal morphology, which was confirmed by IHC staining. These regions were selected, in each section, by a board-certified pathologist (Fig. 1a–d). In the case of BPH, only glandular tissue was highlighted (Fig. 1e–h). Subsequently, 10 mm^2^ of each region were marked to dissect with LCM system (Nonn et al. 2010; Hoefig and Heissmeyer 2010) (PALM Robo, Zeiss, Germany). Optimization assays indicated that Laser Pressure Capture mode (Auto-LPC) alone with non-membrane slides is sufficient for the dissection of tissues for further analysis. LCM was performed under following conditions: LCP energy—80–90, LCP spot distance—25 μm, magnification—5 ×, tissue collected in 20 μl of Digestion Buffer (RecoverAll, Ambion, Thermofisher) in 500 μl sterile PCR-tube cap. Each LCM was preceded by optimization of LCP energy and spot distance to provide a full dissection of marked areas. Caps were sealed back with tubes, centrifuged briefly and placed on wet ice until further steps.

### RNA isolation

Since FFPE treated nucleic acid are degraded and presence of protein crosslinks (Evers et al. 2011) hinder proper extraction, RecoverAll for FFPE kit (Ambion, Thermofisher, USA) was chosen for further analyses on the basis of prior optimization (Suppl. Figure 1). Total RNA extraction was conducted according to the manufacturer guidelines (100 µl Digestion Buffer volume and skipped deparaffinization). RNA was eluted with 60 µl ultrapure, molecular-grade water and stored in − 80 °C until further steps (Paskal et al. 2018).

### RT-qPCR

Extracted RNA was quantified with NanoDrop 2000 spectrophotometer (Thermo Fisher Scientific, USA) with an assessment of A260/A280 (min > 1.7). 100 ng of RNA was used for reverse transcription assay (TaqMan^®^ MicroRNA Reverse Transcription Kit, Thermofisher) with primers for snU6, RNU43, miR-96-5p, miR-134-5p, miR-181b-5p and miR-200b-3p (TaqMan^®^, Thermofisher). cDNA from previous reactions was used for quantitative Real-Time PCR (qPCR). qPCR was set up as it follows: 2 µl cDNA, 7,5 µl qPCR master mix SensiFAST Probe Lo-Rox (BIO-84020, Bioline, UK), 0,5 µl microRNA-specific TaqMan assays snU6, RNU43, hsa-miR-96-5p, hsa-miR-134-5p, hsa-miR-181b-5p, hsa-miR-200b-3p (Assay no.: 001973, 001,095, 000,186, 001,186, 001,098, 002,251) (TaqMan^®^, Thermofisher, USA); molecular-grade water to final volume: 15 µl. U6 and RNU43 expression was used for miRNAs normalization between samples. Reactions were performed in triplicates. qPCR reaction was performed on Applied Biosystems^®^ 7500 Real-Time PCR System (Thermofisher, USA) with the following setup: 1 × 95 °C 5 min, 45 × cycles 95 °C 10 s and 60 °C 50 s.

### Data processing and analysis

Data were collected and processed with Excel 2016 (Microsoft, USA). Statistical analyses were conducted with GraphPad Prism 8.4.3 (GraphPad Software Inc.) using the following tests: Mann-*U*-Whitney, Chi-square, *R*-Spearman correlation coefficient, Wilcoxon signed-rank test. A *p* value of < 0.05 was considered statistically significant. The heatmap was generated using Graphpad using the − Δ*C*t values and *Z*-score. ROC curves analysis was calculated in GraphPad Prism.

## Results

**Fig. 2:**
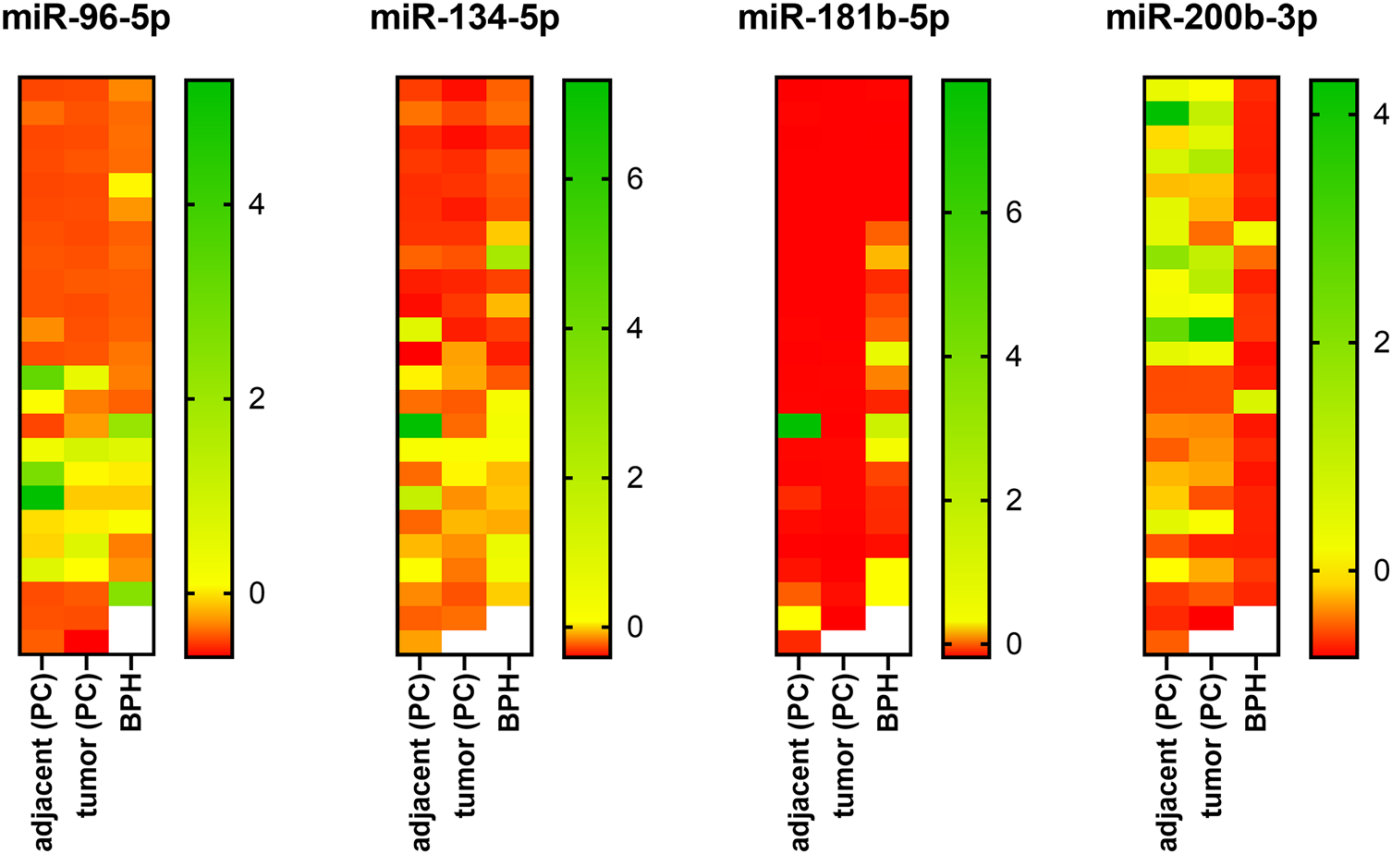
Relative expression of miR-96-5p, miR-134-5p, miR-181b-5p, miR-200b-3p in tumor tissue of prostate cancer (PC), adjacent unaffected tissue and in patients with benign prostatic hyperplasia (BPH). Relative expression is depicted as *Z*-score of relative expression (log10 of 2^−ΔCt^). Red represents the lowest expression, green represents the highest expression

The expression of miR-96-5p, miR-134-5p, 181b-5p, and miR-200b-3p was analysed in 23 PC and 22 BPH samples. From each FFPE sample of PC, tumor tissue and adjacent morphologically healthy tissue were dissected using Laser Capture Microdissection (Fig. 1). In BPH, only glandular tissue was dissected from each FFPE sample. We found that the microRNA expression profile of BPH differs from PC (Fig. 2).

**Fig. 3:**
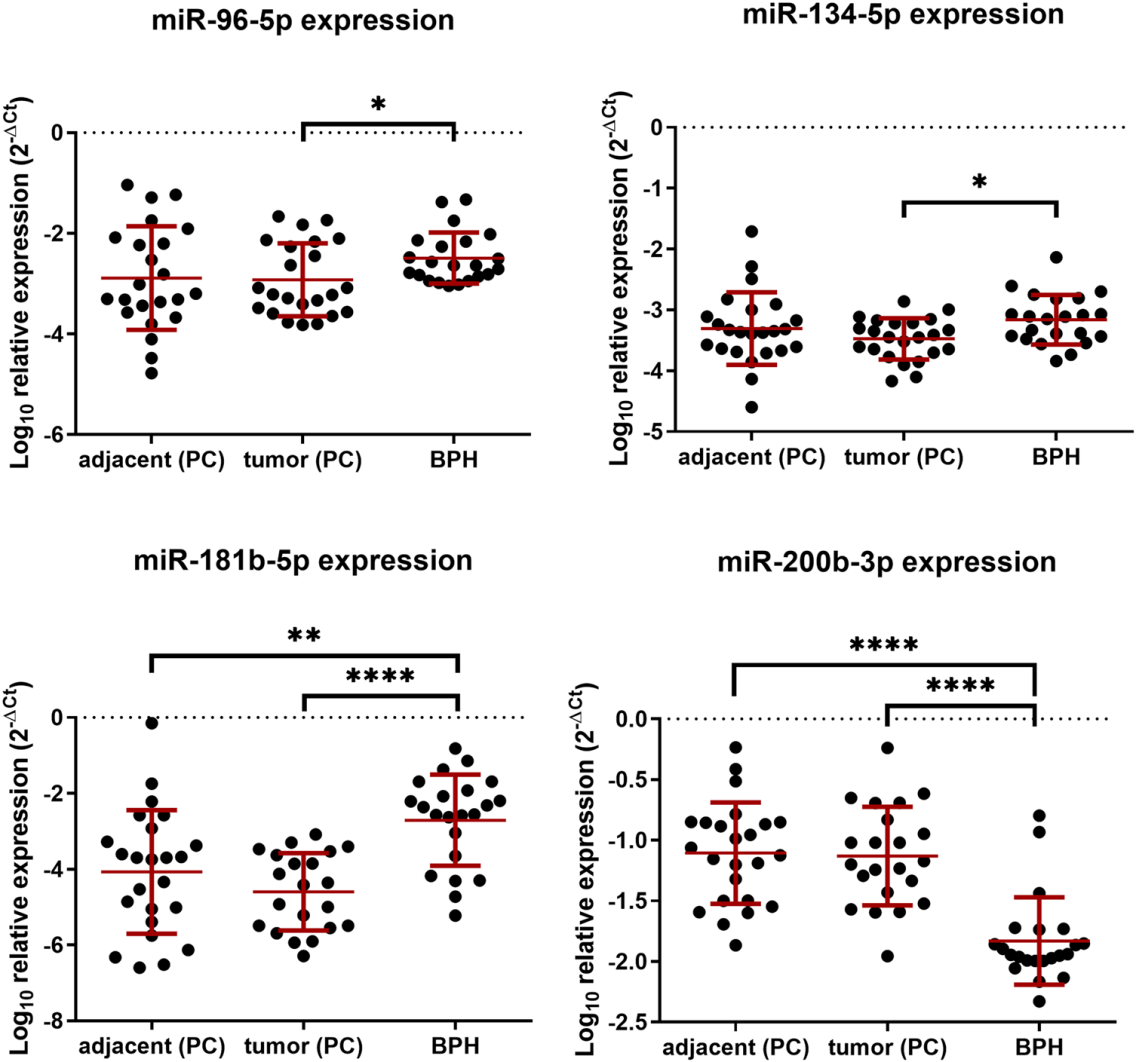
Relative expression of miR-96-5p, miR-134-5p, miR-181b-5p, miR-200b-3p, depicted as log10 of 2^−ΔCt^. Each of prostate cancer (PC) sample has had dissected tumor cells (tumor) and adjacent, unaffected tissue (adjacent). Benign prostatic hyperplasia (BPH) samples have had glandular tissue alone dissected. Tumor and adjacent samples’ expression was tested with the Wilcoxon signed-rank test, the difference between tumor/adjacent and BPH was tested with Mann-*U*-Whitney test. *p < 0.05, **p < 0.01, ***p < 0.001, ****p < 0.0001

In PC the expression of miR-96-5p and miR-134-5p was downregulated compared to BPH (fold change 3.78, *p* = 0.0257; fold change 2.09, *p* = 0.0111, Fig. 3). miR-181b-5p was downregulated 93 times in PC and 19 times in adjacent tissue samples compared to BPH samples (*p* < 0.0001 and *p* = 0.0014, respectively). Moreover, there was a reverse correlation between miR-96-5p, miR-134-5p, 181b-5p expression and Gleason score (Table 2). No other parameter (age, lymphadenectomy, TNM) significantly correlated with any of examined microRNAs’ expression (*p* < 0.05, data not shown). Conversely, miR-200b-3p was upregulated nearly six times in PC samples compared to BPH and seven times compared to adjacent tissue (*p* < 0.0001 and *p* < 0.0001, respectively).

**Table 2:** Results of R-spearman correlation coefficient of Gleason score and miR-96-5p, miR-134-5p, miR-181b-5p, miR-200b-3p expression

*R*-Spearman correlation coefficient of Gleason score and mirs expression				
	miR-96-5p	miR-134-5p	miR-181b-5p	miR-200b-3p
Gleason score	− 0.599*	− 0.517*	− 0.448*	0.259

Gleason score was summed up to obtain interval data

*p < 0.05

Despite precise dissection of tumor tissue and histologically healthy tissue, we did not observe any differences in miR-96-5p, miR-134-5p, miR-181b-5p and miR-200b-3p expression between tumor and adjacent tissue (Fig. 3).

**Fig. 4:**
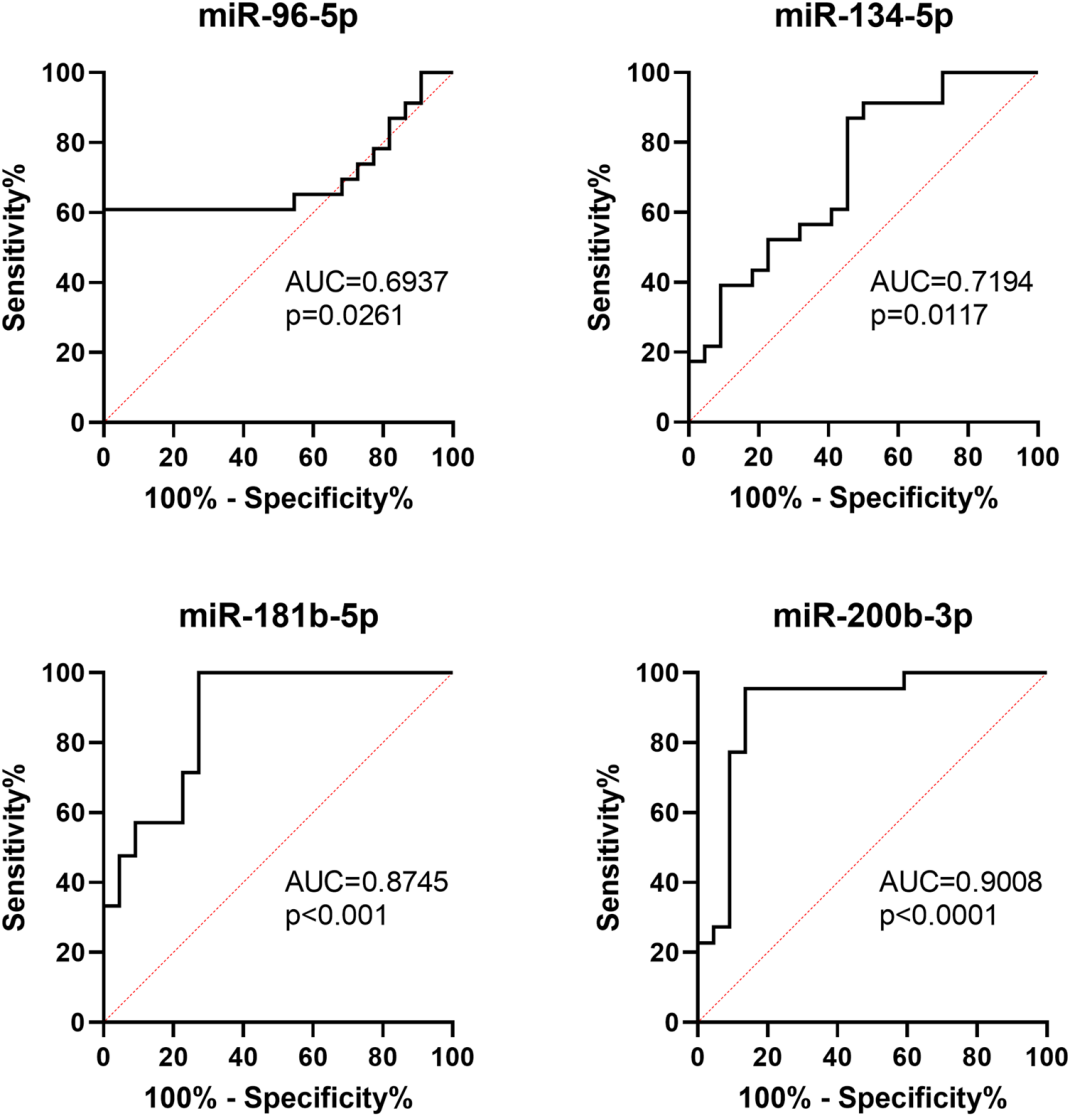
ROC curves analysis for distinction of prostate cancer from benign prostate hyperplasia based on single microRNA expression

**Fig. 5:**
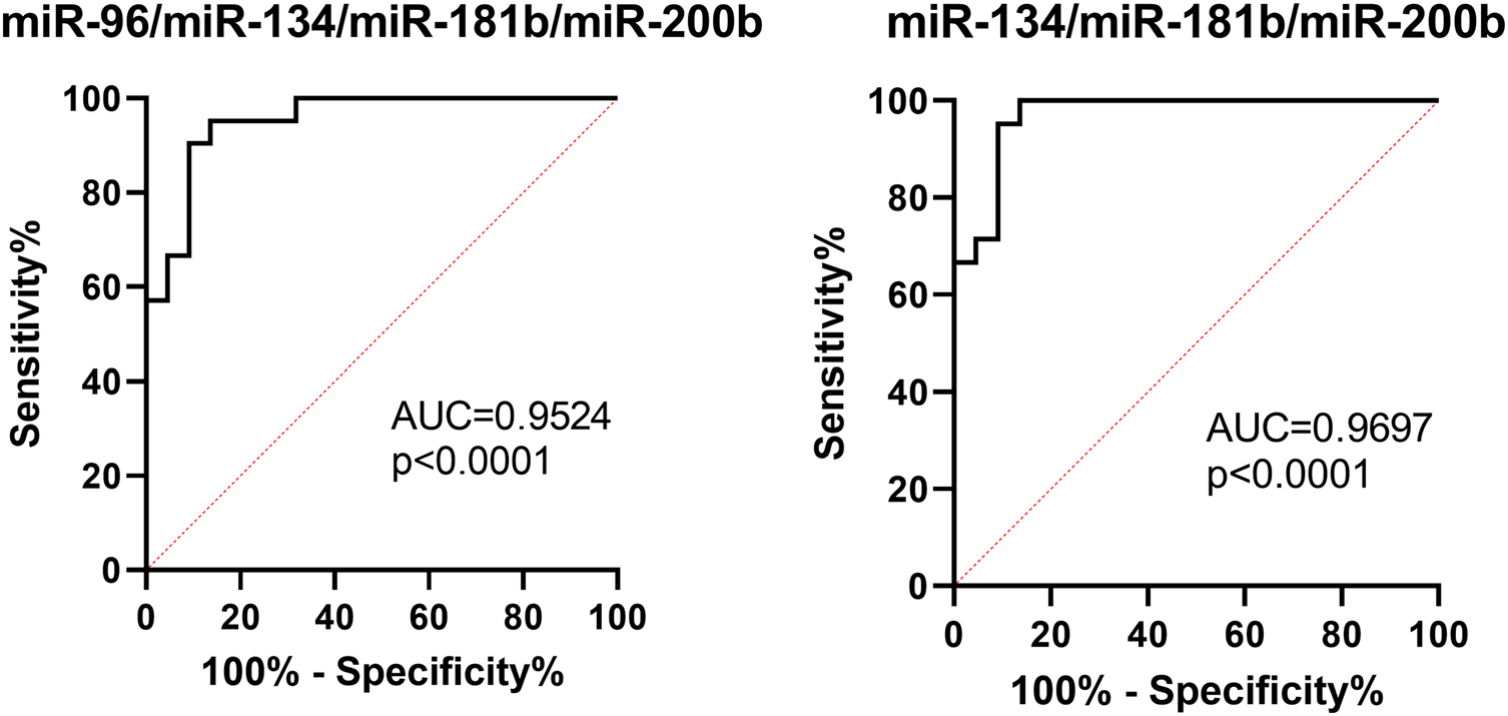
ROC curves analysis for distinction of prostate cancer from benign prostate hyperplasia based on combined microRNA expression including panel of four miRNAs (4-miR) and three miRNAs (3-miR)

To analyse the possible utility of investigated miRNAs in differential diagnosis between PC and BPH, we performed ROC curves analysis (Fig. 4). From four analysed miRNAs, the expression of miR-200b-3p was the most specific and sensitive indicator of PC (AUC 0.9008 (95% CI 0.7987–1.000), *p* < 0.0001). Log_10_ relative expression of miR-200b-3p higher than − 1.658 favours diagnosis to PC than BPH with sensitivity 95.45% and specificity 86.38%. Analysis of combined expression of more than one miRNA revealed that all four miRNAs may be used to support differential diagnosis. For four-miRNA panel (4-miR), log_10_ relative expression was used according to the Eq. 4—miR = − [miR-96-5p + miR-134-5p + miR-181b-5p – (5 × miR-200b-3p)]. 4-miR was characterized by high AUC (0.9524, 95% CI 0.8946–1.00, *p* < 0.0001). For values lower than − 1.287, sensitivity was 90.48% and specificity 90.91% (Fig. 5). Panel of three miRNAs (3-miR) was calculated according to the Eq. 3—miR = − [miR-134-5p + miR-181b-5p – (5 × miR-200b-3p)] was characterized by AUC = 0.9697 (95% CI 0.9259–1.000, *p* < 0.0001) and sensitivity 95.24% and specificity 90.91% for values lower than 1.243 (Fig. 5).

## Discussion

Routine core needle biopsies of prostate tumor consist of both cancer cells and adjacent non-cancerous prostate tissue. Such specimens are often used in RNA expression studies. Substantial contamination of RNA derived from non-cancerous cells may significantly alter the miRNA expression pattern. Thus, in this study, we dissected either PC tissue or adjacent, apparently healthy prostate gland. We compared the expression of given microRNA in PC tissue or adjacent healthy tissue in one prostate specimen. Our study was limited to four miRs of explicit expression in PC, basing on aforementioned studies.

We found that low expression of miR-200b-3p and the high expression of miR-181b-5p may favour the diagnosis towards BPH. Panel of combined expression of miR-134-5p, miR-181b-5p, and miR-200b is a promising tool to support differential diagnosis between PC and BPH. We found that expression of these four microRNAs remains comparable in cancer and non-affected tissue in PC samples. It suggests that changes of the microRNA expression profile are not limited to cancer cells, but also include adjacent, morphologically non-affected tissues. Multiple tumor-secreted microRNA were reported to modify tumor microenvironment (Pan et al. 2020), for instance to reprogram fibroblasts to become cancer-associated fibroblasts (Mitra et al. 2012). The diffusive feature of microRNA expression in tissues was firstly reported by Levine et al. (Levine et al. 2007). Further consideration and studies confirmed that microRNA may diffuse from tumor and act as tumor frontline invasion mediators (Vasilescu et al. 2020). All above explains the reported presence of similar microRNA expression pattern in both tumor and tissue within close distance to the tumor that was histologically unaffected. On the other hand, the design of our study did not include an additional external control of intrapatient microRNA expression pattern, e.g., different type of tissue or blood sample. Such analysis could reveal if microRNA expression pattern was patient-leaned and may have influenced the results. To minimize the effect of the origin of a samples, we employed proper statistical analysis for dependent (PC tumor vs adjacent) and for independent samples (PC vs BPH). Also, as it is depicted on heatmap, (Fig. 2) in case of miR-96-5p, miR-134-5p and miR-200b-3p, we observed that 1/3 to 1/2 of samples had highly similar expression in tumor vs adjacent tissue. Higher number of samples could reveal if the phenomenon is patient-dependent, disease-dependent or microRNA target-dependent. Conversely, miR-181b-5p was evenly expressed in PC-derived groups.

In recent years, several studies showed the clinical significance of miRNAs in PC pathogenesis. Most of the microRNAs act as tumor suppressors and are downregulated in PC cancer cells, e.g., Let-7 family, miR-221, miR-200a (Sequeiros et al. 2013). On the other side, some miRNAs are potently overexpressed in PC and promote tumor development (Sequeiros et al. 2013).

miR-96-5p promotes prostate cancer cells proliferation by targeting tumor suppressor gene FOXO1 (Yu et al. 2014b; Haflidadottir et al. 2013; Fendler et al. 2013). Xu et al. showed that miR-96-5p promoted colony formation, proliferation, and invasiveness of PC cells by targeting MTSS1 (Xu et al. 2016). Another study presented a mechanism in which epidermal growth factor receptor (EGFR) induced the expression of miR-96-5p. It targeted ETV6, the tumor suppressor, which leads to PC progression (Tsai et al. 2017). Moreover, miR-96-5p regulated autophagy under hypoxia in PC cells by targeting mTOR or ATG7 (Ma et al. 2014). It was shown that miR-96-5p modulates androgen signalling (Long et al. 2019) and takes part in prostate bone metastasis formation (Siu et al. 2015). However, its role in PC pathogenesis and interaction between PC cells and tumor microenvironment is complex and need to be further investigated. Several studies showed upregulation of miR-96-5p in prostate cancer tissues (Mihelich et al. 2011; Yu et al. 2014b; Haflidadottir et al. 2013; Navon et al. 2009; Larne et al. 2013). On the contrary, Kang et al. did not observe a correlation between the level of miR-96-5p expression and any clinicopathologic parameter (Kang et al. 2012) while in our cohort, levels of miR-96-5p negatively correlated with Gleason score. Walter et al. showed downregulation of miR-96-5p in high-grade PC tumors, what stays in line with our findings that, we found miR-96-5p showed lower expression in PC compared to BPH (Walter et al. 2013a).

So far, little is known about the role of miR-134 in PC biology. Presented data suggest the tumor suppressive role of miR-134-5p in human cancers. By targeting various genes, it influences oncogenic signalling pathways, e.g., MAPK/ERK pathway, Notch pathway, and EGFR. Upregulated-miR-134 inhibits the expression of cyclin D/cyclin D2/CDK4, KRAS, EGFR, POGLUT1, and STAT5B thus decreases cells’ proliferation. Since miR-134 targets and inactivates KRAS, Nanog mRNA, HNF4α, EGFR, ITGB1, and FOXM1, it also inhibits tumor invasion and metastasis (Pan et al. 2017). Ngalame et al. showed the negative correlation of miR-134 with RAS oncogenes. Downregulation of miR-134 led to the activation of RAS/ERK and PI3K/PTEN/AKT signalling pathways in human prostate epithelial and stem cells (Ngalame et al. 2014). Our study showed the downregulation of miR-134-5p in prostate cancer compared to BPH and negative correlation with Gleason score.

Tong et al. reported overexpression of miR-181 in prostate cancer. In this study, miR-181 promoted cells proliferation and tumor growth in mice via targeting DAX-1, a negative regulator of androgen receptor in PC (Tong et al. 2014). DAX-1 inhibits aromatase expression (Lanzino et al. 2013), but its role in PC cancer rather relies on modulation of carcinogenesis than sex-steroids mediated pathway (Nakamura et al. 2009). Analysis of RNA circularization in localized PC demonstrated that circCSNK-1 interacted with miR-181 and promoted cell growth (Chen et al. 2019).

In our study, expression of miR-181b-5p was significantly lower in PC and adjacent tissue compared with BPH. Moreover, the level of expression of miR-181b-5p correlated with Gleason score.

Several studies show the tumor-suppressive role of miR-200b in PC by targeting different genes, e.g., ZEB1, ZEB2 (Kong et al. 2009; Williams et al. 2013) and Bmi-1 (Yu et al. 2014a). ZEB1/2 proteins are hallmarks of epithelial-mesenchymal transition (EMT) and cofactors chemoresistance in PC (Orellana-Serradell et al. 2019) while BMI1 promotes cell proliferation, EMT and is critical for the development of castration-resistance in PC (Zhu et al. 2020). Katz et al. showed a link between low expression of miR-200b, the Gleason score as well as shorter survival (Katz et al. 2014) and pointed at its possible role as a potential prognostic marker. On the other hand, overexpression of circulating miR-200b in plasma was associated with bone metastasis, high PSA and bilateral tumor (Souza et al. 2017). MiR-200b was downregulated in PC tissue compared with healthy tissue and in PC cell lines compared to normal epithelial prostatic cells in the study of Yu et al. (Yu et al. 2014a). On the contrary, Hart et al. revealed upregulation of miR-200b in samples of PC (Hart et al. 2014). Moreover, in our previous study, we have shown that expression of miR-200b was higher in PC than in BPH samples (Janiak et al. 2017). In this study, we demonstrate that miR-200b-3p is downregulated in BPH samples compared to PC tissue but also to the adjacent, morphologically healthy tissue. These controversies between molecularly confirmed suppressive role of miR-200b and clinically opposite observations raise question what is the source of miR-200b in examined tissues—PC or invaded tissues? Explanation requires further research in spatial context of tissues.

miRs may be analyzed in tumor tissues as well as in body fluids. Exosomal PC-derived miRs are intensively researched as they seem to appear as more stable and promising non-invasive biomarkers of PC (Moustafa et al. 2018; Brase et al. 2011). Several studies showed the potential of microRNAs in urine or blood as diagnostic markers to discriminate PC from BPH (Haj-Ahmad et al. 2014; Al-Kafaji et al. 2018; Cochetti et al. 2016). Haj-Ahmad et al. showed different expression of miR-1825 and miR-484 in urine samples from healthy males and patients with BPH which may be valuable for PC and BPH differentiation (Haj-Ahmad et al. 2014). Although many studies analyzed the role of microRNAs in PC, the data vary and there is a need for further investigation (Sharma and Baruah 2019). In our opinion, assessment of miR-200b-3p and miR-181b-5p levels in blood and urine of PC and BPH patients may be a non-invasive diagnostic approach that is worth further studies.

Discordant results of various studies together with our findings indicates the need for further large-scale studies to answer whether indeed low level of miR-200b-3p or high level of miR-181b-5p indicates BPH. Moreover, our panel of combined expression of three or four miRNAs requires verification on larger cohort of PC and BPH patients along with correlation with clinical data.

## Conclusions

miR-200b-3p expression was higher and miR 181b-5p was lower in PC tissues in comparison with BPH. miR-96-5b and miR-134b-5p are downregulated in PC compared with BPH. Thus, these microRNAs may differentiate BPH and PC. Further studies are needed to assess the clinical usefulness of these microRNA. There are no differences between levels of miR-96-5p, miR-134-5p, miR-181b-5p and miR-200b-3p in prostate cancer and adjacent tissue. miR-96-5p, miR-134-5p and miR-181b-5p correlate negatively with the Gleason score.

## Electronic supplementary material

Below is the link to the electronic supplementary material.

Supplementary file1 (DOCX 89 KB)
