# Supplementary material for: miR-96-5p, miR-134-5p, miR-181b-5p and miR-200b-3p heterogenous expression in sites of prostate cancer versus benign prostate hyperplasia—archival samples study
Source: Histochem Cell Biol. 2020 Dec 17;155(3):423–33. doi: 10.1007/s00418-020-01941-2 (PMC8021536; doi:10.1007/s00418-020-01941-2)
Supplement: Supplementary file 1 — Supplementary file1 (DOCX 89 KB) [file 418_2020_1941_MOESM1_ESM.docx]

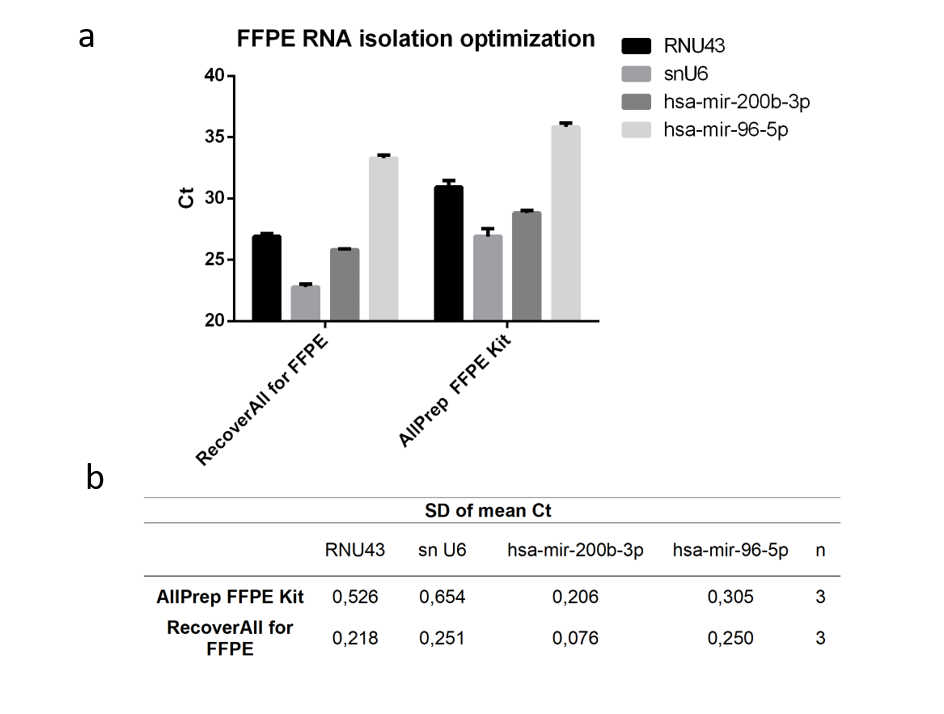


Supp. Fig 1 Preservation of a sample with FFPE techniques results in decreased quality of nucleic acids. Thus, fine isolation protocols are implemented to extract DNA or RNA (especially miRNA) of satisfactory quality and quantity. Primary attempts of RNA isolation from LCM tissues relied on organic extraction with TRIzol® (Thermofisher, USA), which was proven to be efficient in low input FFPE tissue by other researchers. The protocol was modified to focus on miRNA fraction and was preceded with proteinase K digestion. Dissatisfactory yields and quality of RNA required further optimization. Comparison of two silica-column based kits for miRNA extraction from FFPE samples after LCM (10mm^2^). two silica-column based kits were compared – RecoverAll for FFPE (Ambion, Thermofisher, USA) and AllPrep FFPE (Qiagen, USA). RecoverAll kit was chosen for further analyses due to lower Ct of targets and lower variances among 3 identical samples as shown in the Figure; a. Cycle threshold (Ct) in qPCR reaction for RNU43, snU6, miR-200b-3p and miR-96-5p extracted with RecoverAll or with AllPrep from 3 identical samples. b. SD of means Ct for the targets from 3 identical samples extracted with both kits.
